# Comparative Analysis of *Gracilaria chouae* Polysaccharides Derived from Different Geographical Regions: Focusing on Their Chemical Composition, Rheological Properties, and Gel Characteristics

**DOI:** 10.3390/gels10070454

**Published:** 2024-07-11

**Authors:** Xiong Li, Wanzi Yao, Cebin Hu, Congyu Lin, Lijun You, Jian Mao

**Affiliations:** 1 Southern Marine Science and Engineering Guangdong Laboratory (Guangzhou), Guangzhou 511458, China; boyyerli@163.com (X.L.); maojian@jiangnan.edu.cn (J.M.); 2 School of Food Science and Engineering, South China University of Technology, Guangzhou 510640, China; yaowanzi@stu.pku.edu.cn (W.Y.); hghucb@163.com (C.H.); 3 Department of Food Safety and Health, School of Advanced Agricultural Sciences, Peking University, Beijing 100871, China; 4 School of Food Science and Technology, Jiangnan University, Wuxi 214122, China; lincongyu@jiangnan.edu.cn

**Keywords:** *Gracilaria chouae*, rheological property, gels, TPA, anhydrogalactose

## Abstract

Polysaccharides derived from diverse sources exhibit distinct rheological and gel properties, exerting a profound impact on their applicability in the food industry. In this study, we collected five *Gracilaria chouae* samples from distinct geographical regions, namely Rizhao (RZ), Lianyungang (LYG), Ningde (ND), Beihai (BH), and a wild source from Beihai (BHW). We conducted analyses on the chemical composition, viscosity, and rheological properties, as well as gel properties, to investigate the influence of chemical composition on variations in gel properties. The results revealed that the total sugar, sulfate content, and monosaccharide composition of *G. chouae* polysaccharides exhibit similarity; however, their anhydrogalactose content varies within a range of 15.31% to 18.98%. The molecular weight distribution of *G. chouae* polysaccharides ranged from 1.85 to 2.09 × 10^3^ kDa. The apparent viscosity of the LYG and BHW polysaccharides was relatively high, whereas that of RZ and ND was comparatively low. The gel strength displayed a similar trend. BHW and LYG exhibited solid-like behavior, while ND, RZ, and BH demonstrated liquid-like characteristics at low frequencies. The redundancy analysis (RDA) analysis revealed a positive correlation between the texture profile analysis (TPA) characteristics and anhydrogalactose. The study could provide recommendations for the diverse applications of *G. chouae* polysaccharides derived from different geographical regions.

## 1. Introduction

Polysaccharides derived from marine algae have garnered considerable attention due to their diverse biological activities and wide-ranging applications in food, pharmaceuticals, and biotechnology [1,2]. These complex carbohydrates exhibit numerous functional properties, including anti-cancer, anti-inflammatory, anti-photoaging, and gut microbiota regulatory effects, making them valuable in health-related applications [3,4,5,6,7]. Red algae polysaccharides, primarily agar and carrageenan, have been studied for their remarkable gelling properties and potential health benefits [8,9]. Among these red algae, *Gracilaria chouae*, a species of red algae, stands out for its high polysaccharide content. Despite its potential, *G. chouae* remains relatively understudied compared to other red algae, which presents a unique opportunity for pioneering research. In the food industry, *G. chouae* polysaccharides are particularly valued for their ability to form gels, enhance texture, and stabilize emulsions, making them essential in the development of various food products, from dairy to confectionery [10,11].

The chemical composition and functional properties of polysaccharides can be significantly influenced by the geographical origin of the algae [12,13]. Environmental factors such as water temperature, salinity, light intensity, and nutrient availability play critical roles in shaping the molecular structure and biochemical properties of these polysaccharides [14]. For instance, variations in these environmental parameters can lead to differences in the monosaccharide composition, molecular weight distribution, and the degree of sulfation of the polysaccharides, all of which directly impact their gelling behavior, viscosity, and bioactivity [15,16]. This variability necessitates a comprehensive and systematic analysis to understand how different geographical conditions affect the characteristics of *G. chouae* polysaccharides. By elucidating these relationships, researchers can optimize the cultivation and processing conditions to produce polysaccharides with consistent and desirable properties, ensuring their reliable performance in food applications where consistency in texture and gel strength is paramount.

This study aims to conduct a comparative analysis of *G. chouae* polysaccharides sourced from different geographical regions, focusing on their chemical composition, rheological properties, and gel characteristics. By systematically evaluating these parameters, the research seeks to identify how regional differences impact the polysaccharide profiles and their subsequent functional performance in food systems.

## 2. Results and Discussion

### 2.1. Chemical Composition of G. chouae Polysaccharides

The chemical composition of *G. chouae* polysaccharides derived from five different geographical regions is shown in Table 1. The content of total carbohydrates ranged from 52.11% to 60.33%. Among the samples, *G. chouae* polysaccharides from RZ (60.33%) and ND (59.18%) had a relatively higher content of total carbohydrate, while those from LYG (52.11%) had the lowest. This variation indicated a noticeable difference in total carbohydrate content among the *G. chouae* polysaccharides from different locations. The protein content of *G. chouae* polysaccharides from different origins was low, ranging from 0.14% to 0.28%. The content of sulfate varied from 26.81% to 27.55%, showing high levels across all samples with minimal differences. This consistency suggests that *G. chouae* polysaccharides are acidic polysaccharides rich in sulfuric acid esters. The content of anhydrogalactose, an essential component in carrageenan and agar, ranged from 15.84% to 18.98%. The order of content from highest to lowest was: BHW (18.98%), LYG (18.71%), BH (17.22%), ND (16.23%), and RZ (15.84%). It is worth noting that the total sugar content includes anhydrogalactose, as it can also react with phenol sulfuric acid to produce color. However, as shown in Table 1, the total sugar content of *G. chouae* polysaccharides such as RZ and ND, which have a lower anhydrogalactose content, is relatively higher. This observation may be attributed to the fact that the color development of anhydrogalactose measured by the phenol–sulfuric acid method is not as pronounced as that of galactose due to variations in color rendering among different monosaccharides. In addition, the level of 3,6-anhydrogalactose significantly influenced the gelling properties of these polysaccharides. These results highlight the chemical diversity and potential functional differences of *G. chouae* polysaccharides from various regions, providing a basis for further exploration of their applications in the food and biotechnology industries.

### 2.2. Monosaccharide Composition of G. chouae Polysaccharides

The monosaccharide composition of *G. chouae* polysaccharides from different regions included galactose, glucose, xylose, galacturonic acid, and glucuronic acid, as shown in Table 2. Galactose accounted for approximately 90% (87.91~93.17%) of the total monosaccharide content, while the levels of glucose, xylose, galacturonic acid, and glucuronic acid were comparatively lower. Notably, the polysaccharide derived from BHW exhibited a distinctive profile, with significantly higher levels of galactose compared to other polysaccharides and remarkably lower levels of glucose. As previously mentioned, agar and carrageenan derived from the polysaccharide of red algae primarily consist of galactose and anhydrogalactose, which undergo partial or complete hydrolysis to form galactose during the polysaccharide hydrolysis process using trifluoroacetic acid. This resulted in the finding that *G. chouae* polysaccharide is predominantly composed of galactose, as determined by ion chromatography. Xylose and glucose may originate from substances present in the branched chain structure or other polysaccharide molecules, while glucose could also be derived from red algae starch [17,18,19]. In terms of rheological and gel properties, xylose, glucose, and uronic acid might act as impurities to reduce these properties. It has been reported that branched-chain polysaccharides exhibit lower viscosity compared to the equal molecular weight of straight-chain polysaccharides [20]. Consequently, variations in the monosaccharide composition might give rise to discrepancies in their rheological and gel properties.

### 2.3. Molecular Weights of G. chouae Polysaccharides

**Figure 1:**
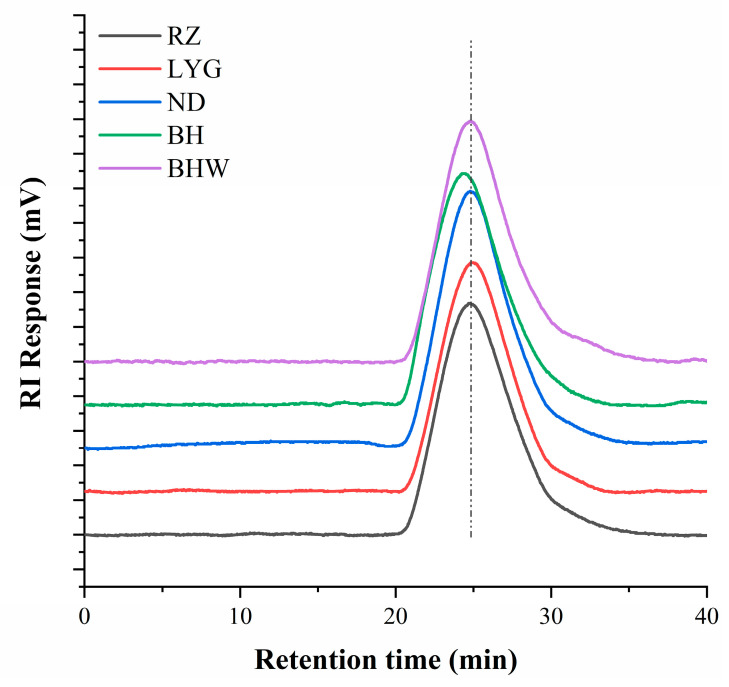
The elution curves of *G. chouae* polysaccharides. The dotted line is the retention time of ND for reference.

The molecular weight distribution of *G. chouae* polysaccharides derived from different geographical regions is shown in Figure 1. The retention time and peak shape of the five polysaccharides exhibited remarkable similarity. The molecular weight distribution of *G. chouae* polysaccharides was considerably high, ranging from 1.85 to 2.09 × 10^3^ kDa. BHW displayed a minimum molecular weight of 1.85 × 10^3^ kDa, while BH demonstrated a maximum molecular weight of 2.09 × 10^3^ kDa. The results showed that the region had little effect on the molecular weight of polysaccharides from *G. chouae*. Despite the fact that a higher molecular weight of polysaccharides leads to increased rheological properties (higher values of G′ and G″) [21], the difference in molecular weight might not be regarded as the primary determinant contributing to the disparity in rheological properties of *G. chouae* polysaccharides due to its negligible magnitude.

### 2.4. The Absolute Viscosity of G. chouae Polysaccharides

**Figure 2:**
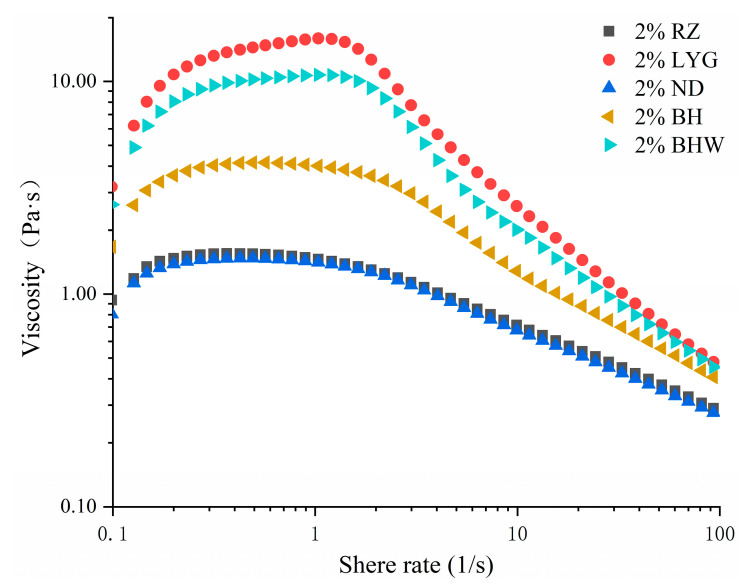
The absolute viscosity of *G. chouae* polysaccharides.

The variation in apparent viscosity is indicative of changes in intermolecular forces, with a higher apparent viscosity corresponding to stronger molecular attractive forces [22]. The flow curves depicting the relationship between apparent viscosity and shear rate are presented in Figure 2. The observed samples exhibited typical shear thinning behavior, characterized by a gradual decrease in apparent viscosity with increasing shear rates (from 0.1 to 100 s^−1^). The observed phenomenon can be ascribed to a reduction in intermolecular interactions among neighboring polymer chains or structural degradation [23]. When the shear rate was 10 s^−1^, the apparent viscosity of LYG polysaccharide exhibited the highest value, while that of ND polysaccharide showed the lowest value at equivalent concentration levels. The viscosity values of *G. chouae* polysaccharides at a shear rate of 10 s^−1^ are presented in Table 3. Among the five *G. chouae* polysaccharides, LYG and BHW polysaccharides demonstrated relatively elevated viscosities, primarily attributed to their higher anhydrogalactose content. The difference in apparent viscosity of *G. chouae* polysaccharides may primarily stem from variations in anhydrogalactose content, as evidenced by the consistent correlation observed between these two factors when considering their chemical composition.

### 2.5. Rheological Characterization of G. chouae Polysaccharides

**Figure 3:**
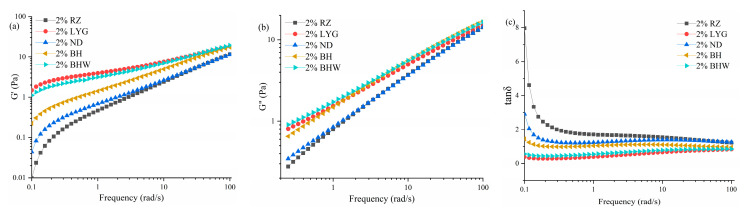
Rheological properties of *G. chouae* polysaccharides. ((**a**) Storage modulus, (**b**) loss modulus, (**c**) tanδ).

An enhanced understanding of the structural properties of polysaccharides from red algae, and their correlation to biological activities, facilitates targeted modifications for enhancing bioactivity or customizing these compounds for specific applications [2]. The storage modulus G′ of a fluid can serve as an indicator of its elasticity, representing the ability of the fluid to recover after deformation, while the loss modulus G″ can provide insights into the viscosity characteristics exhibited by the fluid [24]. The frequency dependence of the moduli of *G. chouae* polysaccharides is illustrated in Figure 3. The storage modulus and loss modulus of BHW, LYG, and BH exhibited relatively high values according to the variation trend of G′ and G″, whereas RZ and ND demonstrated relatively low values at the same concentration. Moreover, the tanδ values of all *G. chouae* polysaccharides tend to approach 1 at high frequencies, indicating the weak gel properties of *G. chouae* polysaccharides (Figure 3c). However, at low frequencies, BHW and LYG exhibited tanδ values < 1, indicating solid-like behavior due to higher storage modulus, whereas ND, RZ, and BH demonstrated tanδ values > 1, suggesting liquid-like characteristics. The aforementioned statement was in accordance with the findings of apparent viscosity.

Polysaccharides exhibit exceptional rheological properties, making them extensively employed in various industrial applications as gelling agents, thickeners, and emulsifiers [25]. The utilization of natural polysaccharides as fundamental constituents in hydrogels fabricated through crosslinked polymeric networks is a common practice [26]. The variation in rheological properties among five types of *G. chouae* polysaccharides at equivalent concentrations suggests potential disparities in gel strength. However, the concentration of the biopolymer is not the sole determinant influencing rheological parameters; rather, factors such as derivatization of the gelling agent, pH adjustment, or the presence of metal ions can significantly modulate mechanical and physical properties [27].

### 2.6. Gel Strength of G. chouae Polysaccharides

The oral behavior of food [28] and the texture of the samples are directly influenced by hardness, springiness, cohesiveness, chewiness, and resilience. Hardness is a quantification of the applied force necessary to induce a specific level of deformation; higher hardness indicates enhanced gel rigidity [29]. Among the *G. chouae* polysaccharides-manufactured gels without any extra ions, BHW and LYG exhibited relatively higher hardness levels, followed by BH, while ND and RZ demonstrated relatively lower hardness, as shown in Table 4. The variation in gel hardness could be attributed to the elevated content of anhydrogalactose within *G. chouae* polysaccharides. This observation was in line with the findings obtained from viscosity and rheology analyses. The springiness refers to the height that the sample recovers during the time interval between the completion of the first cycle and the commencement of the second cycle [30]. Cohesiveness values represent the internal adhesive strength of the sample, and higher cohesiveness is normally correlated with superior gel properties. The springiness of LYG and BHW exhibited higher values compared to ND and RZ, while BH demonstrated intermediate levels among the five materials. In addition, the cohesiveness value of gels ranged from 0.28 to 0.33. The relatively low levels of cohesiveness indicate that the developed gels possess a plastic nature rather than an elastic one, which may be desirable if the gel is intended for easy mastication as a food material [31]. The chewiness refers to the amount of energy needed to masticate a solid or semi-solid sample until it reaches a state that can be easily swallowed [32]. The chewiness value of *G. chouae* polysaccharide gels was similar to that of the springiness value (the chewiness values of LYG and BHW were relatively higher than those of ND and RZ). The resilience values of *G. chouae* polysaccharide gels, however, exhibited slight variations. Specifically, the resilience value of BH was found to be comparable to that of LYG and superior to that of BHW. This difference might be related to the chemical composition of polysaccharides. The palatability of food is determined by two major parameters: texture and flavor. In the case of solid foods, the role of texture is particularly crucial due to the significant textural changes that occur during oral processing, as compared to liquid foods [33]. These results provided references for the application of *G. chouae* polysaccharides in different fields.

### 2.7. RDA Analysis between Chemical Compositions and TPA Characteristics

**Figure 4:**
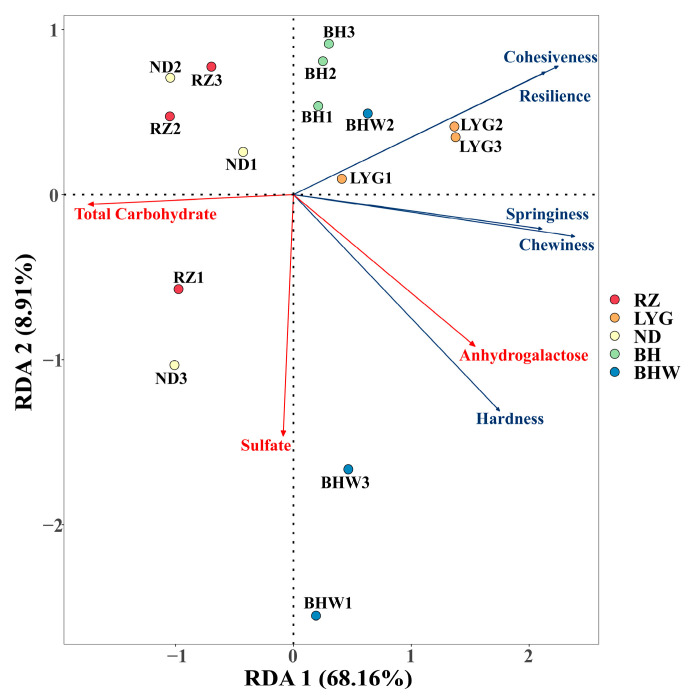
RDA analysis of environmental variables, chemical compositions, and TPA characteristics.

Redundancy analysis (RDA) was an alternative to canonical correlation analysis [34]. The RDA-based approach exhibits a superior combination of low false-positive and high true-positive rates compared to generalized linear models or latent factor mixed models [35]. In this study, the RDA was conducted to further investigate the correlations between chemical compositions and TPA characteristics. As shown in Figure 4, the angle between all TPA characteristics and anhydrogalactose content was acute, indicating a positive correlation. Among these TPA characteristics, the angle between hardness and anhydrogalactose exhibited the smallest value, indicating that anhydrogalactose predominantly influences gel strength. On the contrary, the angle between all TPA characteristics and total carbohydrate content was obtuse, suggesting a discernible negative correlation. As previously mentioned in the chemical composition, a higher total sugar content corresponds to a lower concentration of anhydrogalactose, which exhibits relatively weak color rendering properties compared to other monosaccharides. Consequently, the anhydrogalactose content played a pivotal role in determining the gel’s distinctive features. The sulfate content exhibited a negative correlation with the cohesiveness and resilience of the *G. chouae* polysaccharides. This could be attributed to the inverse relationship between the sulfuric acid group and the mass proportion of the sugar chain, resulting in a reduced number of polysaccharide molecules available for functioning at equivalent masses. Furthermore, both the position and quantity of sulfuric acid groups directly influence the colloidal properties of red algae-derived polysaccharides. For instance, the absence of trivalent ions poses a challenge to achieving gel formation with lambda-carrageenan, which carries three sulfuryl groups attached to every two galactose units [36].

Furthermore, the RDA analysis also demonstrated the resemblance in characteristics among diverse *G. chouae* polysaccharide gels. BH and BHW were distributed in the first quadrant, and ND and RZ were distributed in the fourth quadrant. Similarities between RZ and ND were also observed in the results of apparent viscosity and rheological properties. However, the distribution of BHW was predominantly observed in the second quadrant, suggesting discernible variations in gel properties between polysaccharides derived from wild and artificially cultivated *G. chouae*.

The red algae serve as a crucial source material for the production of galactose and carrageenan, with the gel properties of these compounds being closely associated with the content and configuration of anhydrogalactose, as well as the content and location of connected sulfate groups [37]. In this study, the chemical composition, rheological properties, and gel characteristics of polysaccharides derived from *G. chouae* were investigated to explore the impact of latitude on these polysaccharides. However, no significant correlation was observed between latitude and the aforementioned characteristics of *G. chouae* polysaccharides. Moreover, it is worth noting that the attributes of these polysaccharides may be influenced by factors such as salinity levels, growth cycle variations, and harvest timing within the specific marine region where alga thrives [38,39]. Thus, the investigation of these factors requires further exploration in future studies.

The rheological properties and gelling ability of polysaccharides derived from red algae enable their versatile applications in various fields. On one hand, the high viscosity and rheological properties of red algae polysaccharides make it widely utilized as thickening and gelling agents in the food and cosmetic industries [40]. Concurrently, its exceptional gelling properties confer advantages for application in hydrogels and related materials [41]. Therefore, in industrial applications, alkali extraction is commonly employed to eliminate a portion of sulfate groups while increasing the content of anhydrogalactose to enhance product gel properties. On the other hand, the high molecular weight (MW), intricate structure, elevated viscosity, and gelatinization characteristics of these red algae polysaccharides have imposed limitations on their development and applications [42], especially for their biological activity.

Our previous research has demonstrated that the modification of *G. chouae* polysaccharides resulted in a significant decrease in viscosity, a notable increase in solubility, and an enhancement of microbial accessibility and probiotic activity [43,44]. Therefore, this study can provide improved recommendations for the diverse applications of *G. chouae* polysaccharides derived from different geographical regions. *G. chouae,* sourced from the Rizhao and Ningde regions, emerged as a promising choice for research and application in polysaccharide modification and activity enhancement. In terms of hydrogel preparation and stabilization, *G. chouae* from Lianyungang exhibited distinct advantages, considering the higher cost associated with wild *G. chouae*.

## 3. Conclusions

The present study collected five samples of *Gracilaria chouae* from distinct coastal regions in China. The total sugar, sulfate content, and monosaccharide composition of *G. chouae* polysaccharides exhibit similarity, while their anhydrogalactose content shows significant variation. Moreover, the molecular weight distribution of *G. chouae* polysaccharides exhibits no discernible differences. The apparent viscosity and rheological properties of the Lianyungang and wild sources of Beihai polysaccharides exhibited relatively higher values compared to those of Rizhao and Ningde. The gel strength displayed a similar trend. The TPA characteristics were positively correlated with the content of anhydrogalactose and negatively correlated with the content of total sugar. This study will not only enhance the utilization of *G. chouae* polysaccharides in the food industry but also contribute to the broader field of hydrocolloids and their application in various industries.

## 4. Materials and Methods

### 4.1. Materials and Chemicals

The dried and artificially cultivated *G. chouaes* were procured from Rizhao, Shandong; Lianyungang, Jiangsu; Ningde, Fujian; and Beihai, Guangxi, denoted as RZ, LYG, ND, and BH, respectively. Meanwhile, the wild *G. chouae* purchased from Beihai, Guangxi, was denoted as BHW. Chromatographic-grade monosaccharide standards (including fucose, glucose, arabinose, xylose, galactose, fructose, glucuronic acid, and galacturonic acid) were procured from Sigma-Aldrich (St. Louis, MO, USA).

### 4.2. Preparation of G. chouae Polysaccharides

#### 4.2.1. Pre-Treatment of *G. chouae*

Five types of *G. chouae* were washed to remove impurities and then dried in a drying oven at 60 °C. The dried algae were ground using a universal grinder and passed through a 40-mesh sieve. The resulting *G. chouae* powder was stored in a dry place for future use.

#### 4.2.2. Extraction of *G. chouae* Polysaccharides

A total of 100 g of *G. chouae* powder was accurately weighed and mixed with 5 L of distilled water. The mixture was extracted at 95 °C for 3 h. After extraction, the mixture was filtered through eight layers of gauze to remove residues. The filtrate was then concentrated at 60 °C to half of its original volume using a rotary evaporator (HeiVAP Value, Heidoph, Nuremberg, Germany). Anhydrous ethanol was slowly added to the concentrated solution until the final ethanol concentration reached 60%, with continuous stirring. The mixture was left to precipitate at 4 °C overnight. The precipitate was then centrifuged at 11,971× *g* for 10 min at 4 °C to remove the supernatant (GL21M, Xiangzhi Corporation, Changsha, Hunan, China). The precipitate was washed with 1 L of 95% ethanol, centrifuged again under the same conditions, and the supernatant was discarded. This washing and centrifugation step was repeated once more, followed by a final wash with anhydrous ethanol and centrifugation. The remaining ethanol was evaporated in a fume hood. The precipitate was re-dissolved in a small amount of water and then freeze-dried under vacuum (Alpha80 1-2 LDplus, Marin Christ Corporation, Osterode, Lower Saxony, Germany) to obtain *G. chouae* polysaccharides.

### 4.3. Determination of Chemical Composition

The content of total carbohydrate was determined using the phenol-sulfuric acid method, with glucose as the standard at 490 nm [45]. The content of protein was measured using the Bradford method, with BSA as the standard at 595 nm [46]. The content of sulfate was determined using the barium chloride-gelatin nephelometry method, with K_2_SO_4_ as the standard [47]. The content of anhydrogalactose was determined using the resorcinol method, with fructose as the standard at 554 nm [48].

### 4.4. Determination of Monosaccharide Composition

*G. chouae* polysaccharides (10 mg) were hydrolyzed using 4 mL of trifluoroacetic acid (4 M) at 105 °C for 6 h. The hydrolysates were evaporated and re-dissolved in 4 mL of methanol, then evaporated again. This process was repeated five times to ensure the complete removal of trifluoroacetic acid. The residues were finally dissolved in ddH_2_O and analyzed by ion chromatography ICS 3000 (Dionex Corp., Sunnyvale, CA, USA) with a CarboPac PA20 analytical column (150 × 3 mm; Dionex Corp.), as previously reported [44].

### 4.5. Determination of Molecular Weight Distribution

The molecular weight distribution was determined using high-performance gel permeation chromatography according to our previously reported method [44]. The polysaccharide samples (2.0 mg/mL) were filtered through a 0.45 μm aqueous phase filter, and 25 μL of each sample was injected. The elution was carried out with KH_2_PO_4_ buffer (0.02 M) at a flow rate of 0.5 mL/min. The columns used were TSK-GEL columns (Tosoh Co., Ltd., Tokyo, Japan) in series with a TSK-GEL guard column (6.0 × 40 mm, i.d., 13 μm), G-6000 PWXL (7.8 × 300 mm, i.d., 10 μm), and G-3000 PWXL (7.8 × 300 mm, i.d., 7 μm). The column and detector temperatures (Waters 2414, Waters Corporation, Milford, MA, USA) were maintained at 35 ± 0.1 °C. A standard curve was prepared using dextran standards of various molecular weights.

### 4.6. Dynamic Viscosity and Rheology Analysis

The dynamic viscosity of *G. chouae* polysaccharides was evaluated following our previously reported method [43]. Polysaccharide solutions at a concentration of 20 mg/mL were prepared and equilibrated at 4 °C. The measurements were conducted using a DHR-2 fluid rheometer (TA Instruments Ltd., New Castle, DE, USA) with parallel plates of 40 mm diameter and a gap of 0.5 mm. The shear rate was varied from 1 to 100 s^−1^ at a controlled temperature of 25 °C. Viscosity readings were taken every second over a duration of 60 s. Each sample was tested in triplicate, and the mean viscosity values were recorded for analysis.

The rheological properties were analyzed by dissolving *G. chouae* polysaccharides (20 mg/mL) in distilled water and allowing the solutions to rest at room temperature overnight. Using the same DHR-2 rheometer setup, the storage modulus (G′) and loss modulus (G″) were measured. The shear rate was set to vary from 0.1 to 100 s^−1^ at 25 °C. Triplicate measurements were performed for each sample to ensure reproducibility, and average values were reported.

### 4.7. Texture Profile Analysis (TPA)

The TPA of gels was conducted by first preparing hot solutions of *G. chouae* polysaccharides at a concentration of 40 mg/mL. These solutions were poured into 4.5 cm plastic molds and allowed to cool to form gels. The textural properties of the gels were then assessed using a texture analyzer equipped with a cylindrical probe. The test parameters included a compression distance of 5 mm, a sample compression ratio of 50%, and a probe speed of 1.5 mm/s downward, changing to 1.0 mm/s after contact with the gel. The trigger force was set at 15 g. The maximum force recorded during compression was taken as the gel strength. Additionally, the TPA provided measurements of hardness, elasticity, cohesiveness, chewiness, and resilience. Each gel sample was tested three times, and the average values were calculated and reported.

### 4.8. Redundancy Analysis (RDA)

The relationship between chemical compositions and TPA characteristics was investigated using redundancy analysis (RDA). Prior to the ordination analysis, chemical compositions and TPA characteristics were standardized in order to normalize the distribution of all investigated variables. Among them, protein correlation studies were not conducted due to the limited abundance of protein levels. RDA analysis was performed using the OmicStudio tools at https://www.omicstudio.cn/tool (accessed on 21 June 2024).

### 4.9. Statistical Analysis

The data were expressed as the mean ± standard deviation (SD) of repeated analyses. Statistical analysis was performed using SPSS 19 software (IBM Corp., Armonk, NY, USA). A significance level of *p* < 0.05 was considered statistically significant.

## Figures and Tables

**Table 1:** Chemical composition of *G. chouae* polysaccharides.

	Total Carbohydrate (%)	Protein (%)	Sulfate (%)	Anhydrogalactose (%)
RZ	60.33 ± 0.49 ^c^	0.27 ± 0.01 ^c^	27.08 ± 0.38 ^a^	15.84 ± 0.39 ^a^
LYG	52.11 ± 0.31 ^a^	0.28 ± 0.01 ^c^	27.29 ± 0.30 ^a^	18.71 ± 0.29 ^b^
ND	59.18 ± 0.76 ^c^	0.14 ± 0.01 ^a^	27.55 ± 0.17 ^a^	16.23 ± 0.59 ^ab^
BH	56.04 ± 0.41 ^b^	0.20 ± 0.02 ^b^	26.81 ± 0.08 ^a^	17.22 ± 0.38 ^ab^
BHW	56.26 ± 0.54 ^b^	0.27 ± 0.01 ^c^	27.55 ± 1.02 ^a^	18.98 ± 1.00 ^b^

^a–c^ Means in each column with different superscripts represent significant difference (*p* < 0.05).

**Table 2:** Monosaccharide composition of *G. chouae* polysaccharides (%).

	Galactose	Glucose	Xylose	Galacturonic Acid	Glucuronic Acid
RZ	90.10	4.20	3.56	1.04	1.10
LYG	90.53	3.86	3.92	0.64	1.04
ND	91.36	3.40	3.54	0.72	0.98
BH	88.59	5.74	3.99	0.77	0.91
BHW	93.17	1.27	3.49	0.91	1.17

**Table 3:** Apparent viscosity of *G. chouae* polysaccharides at sheer rate of 10/s.

Samples	RZ	LYG	ND	BH	BHW
Apparent viscosity (Pa·s)	0.72	2.60	0.68	1.29	2.01

**Table 4:** TPA characteristics of *G. chouae* polysaccharides.

Samples	Hardness (g)	Springiness (mm)	Cohesiveness	Chewiness (mJ)	Resilience
RZ	49.76 ± 2.35 ^a^	0.76 ± 0.03 ^a^	0.28 ± 0.01 ^a^	10.46 ± 0.38 ^a^	0.13 ± 0.01 ^a^
LYG	56.18 ± 1.54 ^b^	0.86 ± 0.03 ^b^	0.33 ± 0.02 ^b^	15.95 ± 1.61 ^c^	0.15 ± 0.01 ^b^
ND	50.27 ± 2.25 ^a^	0.77 ± 0.03 ^a^	0.28 ± 0.01 ^a^	10.81 ± 0.89 ^a^	0.13 ± 0.01 ^a^
BH	53.86 ± 0.94 ^ab^	0.79 ± 0.01 ^a^	0.32 ± 0.01 ^b^	13.39 ± 0.11 ^b^	0.15 ± 0.01 ^ab^
BHW	59.66 ± 3.89 ^b^	0.82 ± 0.01 ^ab^	0.30 ± 0.02 ^ab^	14.51 ± 0.33 ^bc^	0.14 ± 0.01 ^ab^

^a–c^ Means in each column with different superscripts represent significant difference (*p* < 0.05).

## Data Availability

The original contributions presented in the study are included in the article, further inquiries can be directed to the corresponding author.
